# Synergistic Coatings Based on Chitosan and *Eugenia caryophyllata* Essential Oil to Improve Postharvest Quality of *Capsicum chinense*

**DOI:** 10.3390/polym18121552

**Published:** 2026-06-22

**Authors:** Fanor David Reyes Pérez, Yeimmy Peralta-Ruiz, Domingo César Carrascal-Hernández, Johannes Delgado-Ospina, Clemencia Chaves-López, Carlos David Grande-Tovar

**Affiliations:** 1 Grupo de Investigación de Fotoquímica y Fotobiología, Universidad del Atlántico, Carrera 30 Número 8-49, Puerto Colombia 081008, Colombia; fdreyes@mail.uniatlantico.edu.co; 2 Programa de Ingeniería Agroindustrial, Facultad de Ingeniería, Universidad del Atlántico, Carrera 30 Número 8-49, Puerto Colombia 081008, Colombia; yeimmyperalta@mail.uniatlantico.edu.co; 3 Grupo de Investigación en Sociedad, Educación y Desarrollo Humano GISEDH, Facultad de Ciencias, Educación, Artes y Humanidades, Institución Universitaria de Barranquilla (IUB), Barranquilla 080002, Colombia; 4 Grupo de Investigación Biotecnología, Facultad de Ingeniería, Universidad de San Buenaventura Cali, Carrera 122 # 6-65, Cali 76001, Colombia; jdelgado1@usbcali.edu.co; 5 Faculty of Bioscience and Technology for Food, Agriculture and Environment, University of Teramo, Via R. Balzarini 1, 64100 Teramo, Italy; cchaveslopez@unite.it

**Keywords:** chitosan, clove essential oil, coatings *P. expansum*, postharvest, sweet chili pepper

## Abstract

The topito pepper (*Capsicum chinense*) is a tropical fruit of economic and gastronomic importance in the Caribbean region, valued for its nutritional content. However, this fruit is susceptible to postharvest fungal diseases, including those caused by the phytopathogenic fungus *Penicillium expansum*, which can degrade fruit quality and pose a health risk due to the potential presence of mycotoxins such as patulin. In this context, we evaluated the protective effects of coatings with chitosan (CS), clove essential oil (CEO), and their combination (CS+CEO) on sweet peppers stored at 12 °C for 12 days after harvest. The results indicate that the film-forming solution exhibited an acidic pH (5.33–5.44), a density of ~1.0 g/cm^3^, and viscosities ranging from 2.75 to 32.9 cP. Furthermore, the results indicate that coatings with CS and CS+CEO significantly reduced weight loss, preserved firmness (19.12–30.40 N), and delayed ripening. At the same time, the coatings exhibited inhibitory effects on *P. expansum* and aerobic mesophiles. The CS+CEO combination demonstrated the greatest inhibitory effect, indicating that it is a sustainable and effective strategy for the postharvest preservation of sweet peppers, thereby enhancing their value, preservation, and food security in the Caribbean region.

## 1. Introduction

Currently, the sweet chili pepper variety topito (*C. chinense*) is one of the most important vegetables in the Caribbean region, where it is cultivated primarily for local consumption and export to international markets. Colombia contributes significantly to production, especially in the Caribbean region, which accounts for the largest share of this variety [1]. However, the topito chili pepper is highly sensitive to microbiological contamination and to post-harvest handling factors, which shorten its shelf life and make it vulnerable to bacterial and fungal infections that compromise its organoleptic quality and commercial value. Several post-harvest diseases caused by phytopathogenic fungi are persistent and pose a difficult management challenge in the production and marketing chain of sweet chili pepper (*C. chinense*), significantly altering quality parameters, including texture, color, aroma, and food safety [2,3].

Among the most persistent postharvest fungal diseases are the phytopathogenic fungi *P. expansum* (which causes blue rot) [4], *Aspergillus niger* (which causes soft rot with black mycelium) [5], *Fusarium* spp. (responsible for watery rots and internal decay) [6], and *Colletotrichum* spp. (the etiological agent of anthracnose), which are characterized by rapidly expanding, sunken necrotic lesions [7]. These pathogens exploit open wounds to infect the fruit, thereby accelerating postharvest quality loss. Due to the high susceptibility of sweet peppers to these fungal infections, it is essential to implement appropriate phytosanitary management and develop effective preservation methods, both of which are priorities for ensuring sustainable production and market competitiveness [8].

In the agrifood industry, numerous post-harvest technologies have been developed to extend the shelf life of highly perishable fruits [9]. Edible coatings made from polysaccharides, lipids, and proteins are among these. Although these materials help delay fruit senescence, they often have limitations, as they do not completely inhibit pathogen development. This is because they are ineffective against infections acquired by fruit before or during the post-harvest stage, thereby reducing their efficacy and restricting their large-scale application in the food industry [10].

In the last decade, several studies have demonstrated the antimicrobial potential of CS (a natural copolymer composed of N-acetylglucosamine (2-(acetylamino)-2-deoxy-D-glucopyranose) and glucosamine (2-deoxy-2-amino-D-glucopyranose) units), which inhibits the growth of certain microorganisms [11]. However, better results have been obtained by combining it with essential oils, which enhance its antifungal activity and inhibit the development of relevant pathogens, such as *P. expansum* strains responsible for blue rot [2]. CEO has proven to be an effective complement to CS due to its high concentration of bioactive compounds with anti-fungal and antioxidant properties [12]. Among these, eugenol (the main component, with strong antifungal and bactericidal action), eugenyl acetate, and β-caryophyllene stand out [13,14]. These compounds act synergistically, altering the permeability of fungal cell membranes and disrupting key metabolic processes [15]. These characteristics make the CS+CEO mixture a promising alternative for prolonging the shelf life of fruits during storage [16].

The implementation of edible coatings in post-harvest handling is projected to be an innovative and sustainable strategy to reduce losses due to fungal diseases and deterioration in climacteric fruits [17]. In this regard, the evaluation of the antifungal effect of emulsions based on CS and CEO on *P. expansum*, as well as their impact on the microbiological, physicochemical, and sensory quality of the topito chili pepper (*C. chinense*), represents a viable alternative to conventional treatments based on chemical fungicides, thus contributing to the development of safer and more environmentally responsible technologies.

In recent decades, a growing number of studies have focused on CS-based coatings enriched with essential oils. Most of these studies have centered on traditional fruits such as apples [18] and tomatoes [19] or on in vitro antimicrobial evaluations, with limited integration of physicochemical, microbiological, and sensory analyses under real-world storage conditions. This study addresses this gap by evaluating the effect of CS and CEO emulsion coatings on the postharvest preservation of *Capsicum chinense*, a crop of regional and gastronomic importance in the Caribbean. Unlike previous studies focused primarily on postharvest preservation, this work establishes a structure–function relationship linking emulsion droplet size distribution to antifungal activity and postharvest stability under real-world storage conditions. Furthermore, integrating in situ fungal studies with physicochemical, microbiological, and sensory analyses provides a comprehensive evaluation framework that more accurately reflects postharvest commercial scenarios. Importantly, the study demonstrates that synergistic interactions between the CS and the CEO enable effective antifungal activity at lower concentrations, thereby improving sensory acceptability and formulation stability.

## 2. Materials and Methods

### 2.1. Fruit Samples

**Figure 1:**
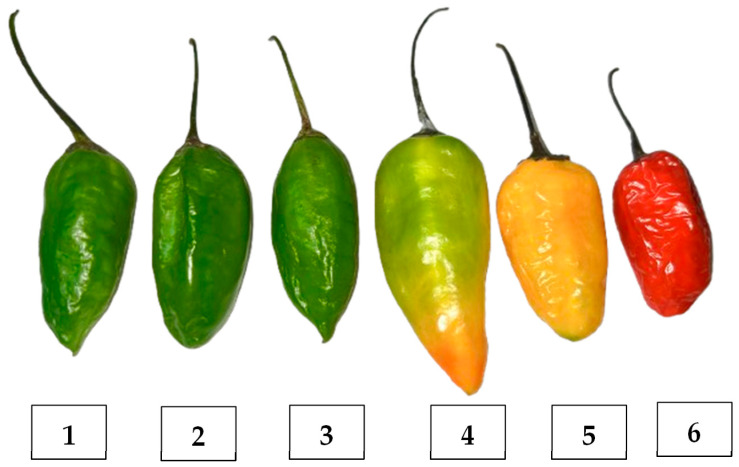
Photographic scale of the different stages of maturation of the topito chili peppers.

Specimens of the topito chili pepper were selected from the Barranquilla wholesale market (10°59′16″ N, 74°47′20″ W), Atlántico, Colombia. Subsequently, the stages were standardized by degree of maturity (stages 1–6), as shown in Figure 1. The maturity scale presented in Figure 1 was adapted from preliminary observations of the fruit’s color and visual characteristics, combined with the general maturity criteria described for *Capsicum* species (according to AGROSAVIA reports). This scale is conceived as a qualitative reference, while only stages 2 and 3 were selected for experimental analyses to ensure the uniformity of the sample (adapted from AGROSAVIA: https://repository.agrosavia.co/items/11aee5fc-ce10-4db6-842c-2bb6765cafda. Accessed on: 30 January 2026). The fruit was cleaned and disinfected with a sodium hypochlorite (NaOCl 200 mg/L) solution, a widely used post-harvest disinfection treatment to reduce microbial contamination and prevent cross-contamination in post-harvest production [20]. NaOCl-based disinfectants are commonly applied at concentrations between 50 and 200 ppm in industrial washing systems and have proven effective in reducing microbial load without compromising fruit quality [21,22,23,24]. These were placed in expanded polystyrene (EPS) trays and then stored in a refrigerator at 12 ± 0.1 °C and 70% relative humidity. Twelve topito chili peppers were periodically selected from each treatment for different analyses.

Photographic scale of the different stages of ripening of the topito chili peppers. Stage 1: completely green fruit; Stage 2: dark green fruit; Stage 3: light green fruit; Stage 4: yellowish-green fruit with initial color change; Stage 5: orange-yellow fruit; Stage 6: fully ripe red fruit.

### 2.2. Identification of the Volatile Compounds of Clove Essential Oil (E. caryophyllata, CEO)

The CEO used in this research was commercially purchased from Marnys (Cartagena, Murcia, Spain) without modification. The composition of the CEO was analyzed by gas chromatography–mass spectrometry (GC-MS) as previously reported [25].

### 2.3. Preparation of Emulsions

The emulsions were prepared according to the most widely documented methodologies [26]. The preparation of the CS emulsion consisted of adding the required amount of CS powder (degree of deacetylation = 75%; Mw = 50,000–190,000 Da, Sigma-Aldrich, Saint Louis, MO, USA) to a 0.1 M acetic acid solution (EMSURE^®^, Darmstadt, Germany) until a solution of 5 mg/mL and pH 5.6 was obtained, under constant stirring. For the preparation of the CS+CEO emulsion, the previous methodology for the CS emulsion was used, adding glycerol (DIY VAPE, Bogotá, Colombia) in a ratio of 2.5 mL/100 mL of CS, as a plasticizer, and was shaken for 30 min; then 10 µL of Tween 80 (Merck^®^, Rahway, NJ, USA) was added as an emulsifier. The concentration of CEO (0.156 µL/mL) was selected based on prior research and preliminary formulation trials to maintain antimicrobial efficacy, emulsion stability, and sensory properties. Previous studies have demonstrated that essential oils rich in phenolic compounds (such as eugenol) exhibit strong antimicrobial and antifungal activities even at low concentrations, particularly when incorporated into polymer matrices such as CS, which act as controlled-release systems, enhancing their effectiveness. Concentrations between 0.1 µL and 1 µL have been widely reported to be effective, minimizing adverse sensory effects and maintaining coating stability [27,28]. In this context, lower concentrations were selected to avoid phytotoxicity, volatility losses, and undesirable aroma alterations. Furthermore, the synergistic interaction between CS and CEO enables the use of lower concentrations while maintaining antimicrobial performance, owing to combined mechanisms of membrane disruption and barrier formation. Therefore, the selected concentration represents a compromise between functional activity and the preservation of fruit quality.

After 1 h, the CEO was added at a concentration of 0.0156% (*v*/*v*; 78 µL of CEO), and the mixture was stirred with a magnetic stirrer (Daihan Scientific, Woungju, South Korea) at 500 rpm for 20 min. For the preparation of the CEO, 500 mL of distilled water was used, with 0.78 μL of Tween 80 (10% of the CEO) added as an emulsifier. After 30 min, the clove essential oil was added. The emulsions were allowed to stand for 72 h at 4 ± 0.5 °C to eliminate any air bubbles.

### 2.4. Application of Edible Coatings to the Topito Chili Pepper

All topito chili peppers were coated by immersion with the different treatments, according to the methodologies reported by Sánchez-González et al. and Peralta et al. [29,30]. The different formulations consisted of a control of chili peppers immersed in distilled water (F1) and chili peppers coated with three formulations (F2 = CS, F3 = CEO 78 µL, and F4 = CS+CEO 78 µL). These were stored in 15 cm × 9 cm × 12 cm glass containers for immersion, which lasted 5 min. Subsequently, they were air-dried for 45 min and stored in polyethylene terephthalate (PET) boxes refrigerated at 12 ± 0.2 °C for 12 days. The evaluations of the topito chili peppers’ physicochemical properties were conducted on days 0, 3, 6, 9, and 12.

### 2.5. Physicochemical Analysis of Emulsions

#### 2.5.1. Analysis of the Droplet Size of Emulsions

Droplet size was determined using a Malvern Panalytical dynamic light scattering analyzer (Malvern, UK) at 25 °C (model ZS Xplorer, DLS technique). Emulsions were diluted 1:100 (*v*/*v*) in the appropriate dispersing phase (distilled water or acetic acid solution for samples containing CS). Each sample was measured in triplicate, and the Z-average (Dh) and polydispersity index (PDI) were recorded from the intensity distribution. The analysis followed the recommendations of ISO 22412 for DLS (www.en-standard.eu).

#### 2.5.2. Density Analysis

The analysis was performed in accordance with the parameters of ISO 8655-2 [31]. For this purpose, a 1 mL LABNET micropipette was used to take each sample. The liquid was then weighed on an Adventurer AR2140 analytical balance, which enabled calculation of the weight Pi and application of the density formula in Equation (1).

(1) $$d=\frac{P_{i}}{V_{i}}$$

where *P*_i_ is the weight of the sample (g), and *V*_i_ is the volume of the sample (1 mL) at 25 °C (g/mL).

#### 2.5.3. Viscosity and pH

The viscosity of the formulations was determined using a Brookfield LVF viscometer (Middleboro, MA, USA). Each sample was placed under the stirrer at low speed until the temperature reached 25 °C ± 0.2 °C; the spin number and speed (rpm) were adjusted in accordance with ASTM D2196-99 (store.astm.org). The pH was determined using a Thermo Fisher Scientific Orion potentiometer (Waltham, MA, USA), calibrated with the Thermo Fisher Scientific Orion calibration kit (4, 7, and 10; Waltham, MA, USA).

### 2.6. Physicochemical Behavior of Coated Topito Chili Peppers

#### 2.6.1. pH

For sample preparation, 10 g of the fruit was macerated with the corresponding treatments and homogenized in 100 mL of distilled water. Subsequently, the pH was measured with a Thermo Fisher Scientific Orion potentiometer, previously adjusted to three standard points: 4.0, 7.0, and 10.0.

#### 2.6.2. Soluble Solids (SS)

For the analysis, 10 g of homogenized pulp were taken. Following the AOAC 932.12 standard (www.aoac.org), the soluble solids content (°Brix) was measured using a BRIXCO-brand portable analog refractometer (LAXCO INC, Broken Arrow, OK, USA), previously calibrated with deionized water at 20 ± 1 °C [32]. pH control and regulation are important aspects of this study, since although CS dissolves in acidic media, small variations in pH significantly influence the polymer’s charge density and, consequently, the coating’s physicochemical stability and antimicrobial performance.

#### 2.6.3. Titratable Acidity

Following AOAC standard 942.15 [33], titratable acidity was determined by potentiometric titration. For this purpose, 10 g of topito chili pepper pulp was mixed with 100 mL of deionized water to which 2 drops of phenolphthalein were added. A 0.1 N NaOH solution, previously standardized [29], was used as the base. The results are expressed as a percentage of citric acid and were calculated using Equation (2):

(2) $$Acidic\;index\;(\%\;citric\;acid)=\frac{V_{1}\times N}{V_{2}}\times K\times 100$$

where $V_{1}$ is the volume of NaOH consumed (mL), $V_{2}$ is the volume of the sample (mL), K is the equivalent weight of citric acid (0.064 g/meq), and N is the normality of NaOH (0.1 meq/mL).

#### 2.6.4. Maturity Index

The maturity index was calculated using Equation (3):

(3) $$Maturity\;index=\frac{\left(\%Brix\right)}{\left(\%\;citric\;acid\right)}$$

where % Brix is the total soluble solids (%) expressed in Brix, and % citric acid is the titratable acid, measured as % citric acid [34].

#### 2.6.5. Weight Loss

Weight loss was calculated by the gravimetric method, considering the difference between the initial weight of the fruit (day 0) in the four treatments and the control with respect to the weight on days 3, 6, 9, and 12 [35], using Equation (4):

(4) $$Loss\;weight\;(\%)=\frac{\left(Pin-Pfin\right)}{Pin}\times 100$$

P_in_ is the initial weight of the samples (g), and P_fin_ is the final weight of the samples (g).

#### 2.6.6. Firmness

To determine fruit firmness, a portable fruit ripeness tester, FT 10 Wagener (model FT. 327) (Greenwich, CT, USA), with an 8 mm diameter plunger was manually inserted into the fruit skin. Penetration force readings were expressed in kilograms-force (kg·f) and converted to newtons (N). Firmness was checked on both sides of each fruit due to its size [36,37].

### 2.7. In Situ Evaluation of the Antifungal Capacity of the Emulsions on the Mycelial Growth of P. expansum

Inoculation was performed using the colonized agar plug method proposed by Sbodio et al. [38]. The fungus was inoculated into the fruit epidermis via an incision; the wounds were opened with a sterile scalpel to a depth of 1 mm and a diameter of 2 mm. Subsequently, CS, CEO, and CS+CEO coatings were applied, and an uncoated negative control was also analyzed. The coated fruits were left to dry in an aseptic laminar-flow chamber for 1 h. On the dried surface, 2 mm of *P. expansum* mycelium, grown for 3 days, was transferred and inoculated into the previously made incisions.

#### Fungal Growth Dynamics with the Treatments

The in situ fungal growth data were analyzed using the Gompertz equation modified by Zwietering et al. [39], with a Python (v.3.14.2) script run locally on Google Colab to estimate the growth parameters: λ (delay time), μ_m_ (maximum exponential growth rate), and A (maximum growth value). Aerobic mesophilic counts were performed according to ISO 4833 (www.iso.org/standard/53728.html, accessed on 16 April 2026), using the plate colony counting technique at 25 °C. Results were expressed as logarithms of colony-forming units (CFU) per gram (Log CFU/g) of mesophilic bacteria.

### 2.8. Microbiological Quality of Coatings

Analyses of the microbiological quality of the coatings were performed in duplicate for aerobic mesophilic counts on days 0, 3, 6, 9, and 12 of the treatments. On these days, topito chili peppers weighing approximately 10 g were collected and homogenized for 10 min in 90 mL of peptonized water. The homogenate was serially diluted from 10^−1^ to 10^−6^, and then 1 mL of each dilution was placed onto Plate Count Agar (PCA) plates and cultured for 48 h.

### 2.9. Determination of the Effect of CS Coatings Incorporated with CEO on the Organoleptic Properties of the Topito Chili Peppers (C. chinense)

The sensory analysis was conducted in accordance with NTC 3932 [40,41]. Fifty untrained panelists were required for the test. They were provided with information on the test methodology. They signed an informed consent form that included details about the reagents used to prepare the emulsions and about any potential allergic reactions they might experience if they are sensitive. Each panelist spent approximately three minutes completing all sections of the form. The test evaluated the attributes of aroma, brightness, color, and texture (repository.agrosavia.co). Panelists were asked to rate the difference between the samples for each attribute, with 0–2 indicating extreme dislike, 3–5 indicating fair, 6–8 indicating good, and 9 indicating excellent [42].

### 2.10. Statistical Analysis

Statistical analysis of the experimental data was performed using analysis of variance (ANOVA) to identify significant differences among the evaluated treatments. Subsequently, Fisher’s LSD (Least Significant Difference) multiple-range test was applied to determine the minimum significant difference between means at a 95% confidence level (α = 0.05). This approach enabled precise discrimination of variations attributable to the different coatings applied to the fruit. The analyses were performed using STATGRAPHICS Centurion XVIII software version 18.1.16. Available online: https://www.statgraphics.com/centurion-xviii (Accessed on 20 January 2026). (www.statgraphics.com).

## 3. Results and Discussion

### 3.1. Identification of the Volatile Compounds of Clove Essential Oil (E. caryophyllata, CEO) by Gas Chromatography Coupled to Mass Spectrometry (GC-MS)

The characterization of the components present in the CEO was carried out by GC-MS, comparing the retention indices and molecular weights with the Adams [43] and NIST05 [44] databases. Table 1 presents all the molecules identified in the CEO [25].

The CEO analysis identified 15 compounds, the most abundant of which were eugenol (76.5%), eugenyl acetate (17.8%), and trans-β-caryophyllene (4.0%), along with α-humulene and caryophyllene oxide (0.5%) [25,49]. These metabolites possess broad biological activities, including antimicrobial, antifungal, anti-inflammatory, and antioxidant properties [50,51], making them promising candidates for microbial and antifungal control. Esters, ketones, terpenes, and phenolic ethers were found in smaller proportions.

The composition of essential oils can vary due to endogenous factors (such as plant age, genotype, population density, and extraction techniques) and exogenous factors (including light, temperature, water, location, and soil) [43,52]. These conditions influence metabolite biosynthetic pathways, generating chemotypes and ecotypes with distinct biological properties, including antimicrobial, larvicidal, and antioxidant activities [43,53].

### 3.2. Physicochemical Characterization of CS Emulsions

Table 2 shows the physicochemical properties of the CS+CEO, CS, and CEO treatments. All maintained an acidic pH (between 5.36 and 5.40), which is typical of formulations containing biopolymers and essential oils. However, significant differences were observed (*p* < 0.05), with the CS emulsion exhibiting the lowest pH. This lower pH causes the free amino groups (-NH_2_) to become protonated, improving the polymer’s ionic stability and solubility, since the pH is below the pKa of the -NH_2_ (6.2–6.6) [54]. This factor is important because the cationic polyamine state of CS influences its colloidal stability and antimicrobial activity, thereby affecting the coating’s efficacy in post-harvest applications. The increased pH of the emulsions (CEO and CS+CEO) may be due to the presence of the essential oil and the surfactant.

The density values ranged from 1.0043 to 1.0087 g/cm^3^, with no significant differences (*p* > 0.05), indicating that the addition of CEO at low concentrations does not alter the apparent density of the emulsion, consistent with previous reports [55].

The viscosity of the CS emulsion was the highest, attributed to CS’s ability to form three-dimensional polymer networks that regulate flow [54]. Similarly, the CEO emulsion exhibited lower viscosity than the CS emulsion, which may be related to molecular length and the oil’s lipophilic nature. The CS+CEO emulsion exhibited a viscosity similar to that of the CS emulsion, suggesting that the oil does not alter the viscoelastic properties of the CS due to its adequate dispersion. In this respect, these results are consistent with other studies that analyze CS as the main contributor to viscosity and stability in emulsions [56].

#### Particle Size of Emulsions

Particle size is a key parameter for evaluating the stability of edible emulsions and coatings, as smaller diameters are associated with greater physicochemical stability. Small particles yield a homogeneous distribution and a larger surface area, which improve interactions with the polymer matrix and barrier properties against moisture, gases, and volatile compounds [57]. Furthermore, they reduce coalescence and sedimentation, preventing phase separation during storage [58].

**Figure 2:**
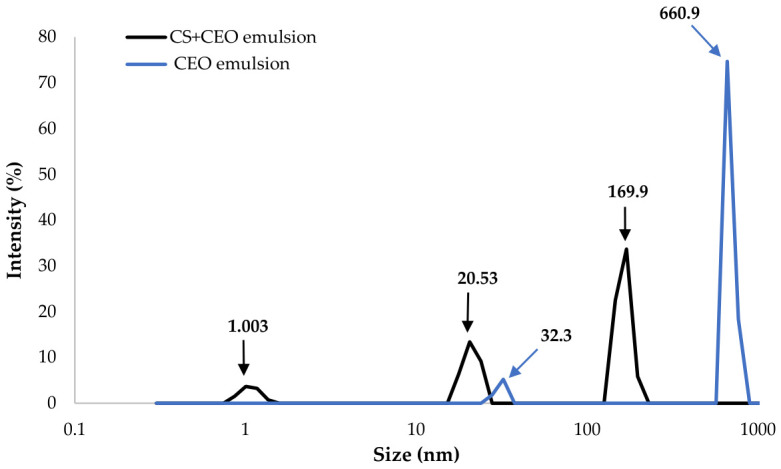
Droplet size distribution in CEO and CS+CEO emulsions.

In essential oil emulsions, reducing the droplet size improves colloidal stability and coating efficacy, thereby extending postharvest life [59]. Recent studies confirm that nanoencapsulation in matrices such as chitosan or proteins maintains homogeneous dispersion and enhances antimicrobial and antioxidant activities [60]. The stability observed in essential oil solutions suggests that the system retains its structural integrity without phase separation, which is favorable for its application in preserving the topito chili pepper (Figure 2).

Dynamic light scattering (DLS) analysis revealed a multimodal distribution in CS emulsions containing clove essential oil, with peaks at 1.0 nm, 20.5 nm, and 169.9 nm. The first peak is associated with low-molecular-weight, non-encapsulated molecules, while the second (20.5 nm) corresponds to the main colloidal fraction, indicating a stable and homogeneous nanoemulsion [61]. The third peak (169.9 nm) represents larger aggregates, possibly due to coalescence or excess biopolymer, although, being below 200 nm, it is still considered acceptable for colloidal formulations [62].

The presence of nano- and submicron populations reflects controlled heterogeneity, typical of biopolymer-stabilized emulsions. This behavior is consistent with previous studies that associate this distribution with interactions among CS, the surfactant, and hydrophobic compounds, thereby improving the functionality of the active coating [63].

DLS analysis of the clove essential oil emulsion revealed a bimodal distribution with peaks at 32.3 nm and 660.9 nm, the latter being predominant (>70%). The smaller fraction in the nanometer range corresponds to surfactant-stabilized nanodroplets, indicating an effective reduction in interfacial tension and a stable dispersed phase [64]. However, the predominance of large particles indicates a conventional microemulsion with limited colloidal stability, likely due to coalescence or a low surfactant concentration. This result suggests the need to optimize emulsification conditions to obtain smaller particles and improve the stability and functionality of the essential oil [65].

### 3.3. Physicochemical Behavior of Topito Chili Peppers Coated with CS and CEO

#### 3.3.1. Weight Loss

**Figure 3:**
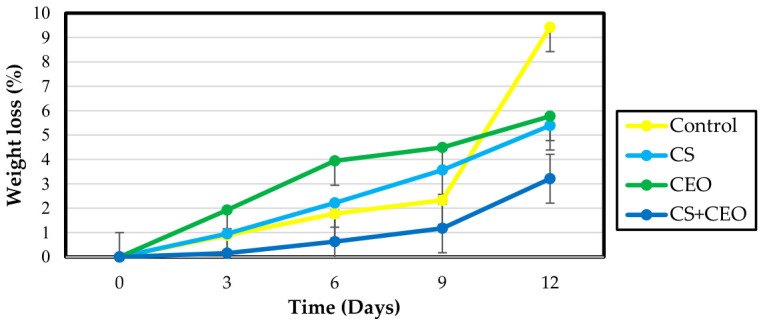
Percentage of weight loss in topito chili fruits (*C. chinense*) during storage under different coatings.

Weight loss in climacteric fruits is primarily due to transpiration and respiration, processes that increase during storage and impair postharvest quality. Transpiration causes water loss and flaccidity, while respiration consumes sugars and organic acids, accelerating senescence [66]. As shown in Figure 3, uncoated fruits exhibited the greatest weight loss (9.42%) after 12 days of monitoring, whereas coated fruits showed a significantly lower loss (*p* < 0.05). This is consistent with studies in other climacteric fruits, where the absence of a barrier promotes rapid dehydration and the loss of respiratory substrates.

Initially, it is important to note that on day 12, there were no significant differences (*p* > 0.05) among the treatments (CS and CEO), which is attributed to the formation of semi-permeable films composed of CS and essential oil. These films limit gas exchange and water vapor transmission, decreasing transpiration and respiration [67]. Thus, the CEO, although with a smaller effect (5.77%), protected its antioxidant and antimicrobial phenolic compounds, which preserve cell integrity [68]. On the other hand, the CS treatment reduced weight loss in the topito chili pepper by 5.39% compared to the control. Interestingly, the CS+CEO combination showed the greatest efficacy (3.21%), demonstrating a synergistic effect between CS and the bioactive compounds in the oil. This coating provides a more efficient physical barrier and improves cell stability by reducing microbial activity and oxidation [69]. Taken together, the results confirm that coatings containing biopolymers and essential oils constitute an effective strategy for extending the shelf life of climacteric fruits, minimizing dehydration and post-harvest quality loss.

#### 3.3.2. Firmness

**Figure 4:**
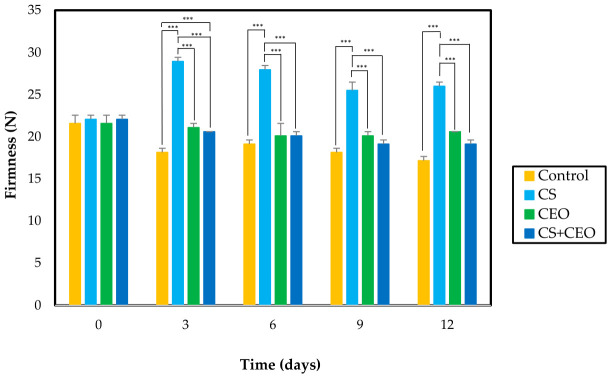
Evolution of firmness (N) of topito chili fruits (*C. chinense*) during 12 days of storage at 12 ± 2 °C, under the different treatments. *** Indicates statistically significant differences at *p* < 0.05.

During storage of the topito chili peppers, a progressive and significant decrease in firmness (*p* < 0.05) was observed across all treatments (Figure 4).

Uncoated chili peppers exhibited reduced firmness, indicating their susceptibility to turgor loss and cellular degradation during ripening [70]. In contrast, fruits coated with CS maintained the greatest firmness over 12 days, with significant differences (*p* < 0.05) compared to the other treatments. This is due to the formation of a semi-permeable film that reduces transpiration and respiration, delaying enzymatic softening [69,71].

At the end of storage, treatments with CEO and CS+CEO were less effective than CS alone, although CEO helped reduce structural deterioration through its antimicrobial and antioxidant properties. The CS+CEO combination exhibited intermediate firmness, indicating a positive, though not synergistic, interaction between the two compounds. Overall, CS, whether used alone or in combination, is confirmed as the best option for maintaining the firmness of the topito chili pepper and extending its postharvest life.

#### 3.3.3. pH of Coated Topito Chili Peppers

**Figure 5:**
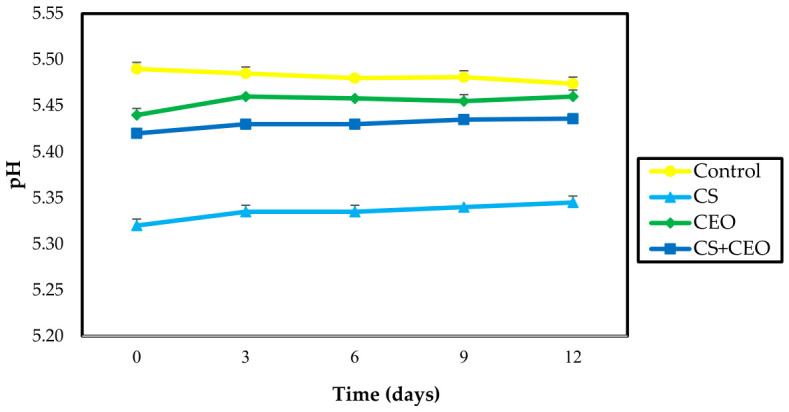
Evolution of pH in topito chili peppers (*C. chinense*) during storage with different coatings.

The pH of climacteric fruits varies during ripening due to the consumption of organic acids during respiratory and metabolic processes. In the topito chili pepper, pH values remained stable across all formulations over 12 days of storage (Figure 5), without significant increases associated with ripening.

This behavior indicates that the coatings helped regulate physiological changes and delay the degradation of organic acids. Fruits treated with chitosan and the CS+CEO combination showed a significantly lower pH (*p* < 0.05) than fruits receiving the other treatments. This is consistent with studies on other CS-coated fruits, in which the barrier effect reduces respiration and transpiration, thus stabilizing the pH [72]. Furthermore, the CEO may have contributed to this stability through its antimicrobial and antioxidant properties, which reduce deterioration and degradation of acidic compounds. Overall, the coatings, especially CS and CS+CEO, proved effective in maintaining pH stability during storage.

#### 3.3.4. Soluble Solids

Table 3 reports the results for soluble solids (°Bx), which remained constant during storage (0 to 12 days) across all treatments evaluated. The control treatment exhibited the highest value (4.00 ± 0.00 °Bx), followed by the CEO treatment (3.50 ± 0.71 °Bx), while the CS and CS+CEO treatments showed the lowest values (3.00 ± 0.00 °Bx). Significant differences between treatments (*p* < 0.05) are indicated by the letter (“a”), where the Control and CEO treatments do not differ significantly from each other. The CS and CS+CEO treatments are in a separate group (“c”), showing significantly lower values compared to the Control and CEO treatments. No significant differences were identified between CS and CS+CEO.

The lack of variability in the Control, CS, and CS+CEO treatments suggests high stability of the parameter, whereas the CEO treatment showed some dispersion (±0.71), indicating greater heterogeneity in the measurements. These findings indicate that the type of treatment significantly influences the soluble solids content, whereas storage time has no significant effect under the evaluated conditions.

The control group exhibited the highest soluble solids value (4 °Brix), followed by the clove essential oil treatment (3.5 °Brix), while CS and the CS+CEO combination remained at 3 °Brix. This stability indicates that the coatings acted as semipermeable barriers, limiting gas exchange and the enzymatic activity that converts starches into simple sugars [73]. Studies on tomatoes and peppers report that CS coatings reduce the accumulation of soluble solids by slowing respiration and carbohydrate degradation. Thus, the lower variation in soluble solids in the treated fruits suggests slower ripening and a longer shelf life. In contrast, the higher value in the control group reflects accelerated ripening, which affects their commercial durability.

#### 3.3.5. Titratable Acidity

**Figure 6:**
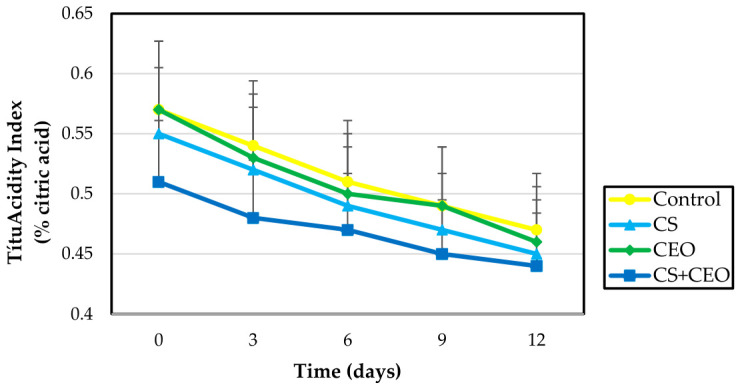
Effect of coatings on the titratable acidity (% citric acid) of topito chili peppers during 12 days of storage at 12 ± 2 °C.

The titratable acidity (TA) of the topito chili peppers decreased across all treatments during storage (Figure 6), a trend associated with the use of organic acids as respiratory substrates during ripening.

Initial values (day 0) corresponded to a coating time of approximately 1 h. This behavior coincides with that reported in other climacteric fruits, in which decreases in citric, malic, and ascorbic acids are associated with reduced acidity and increased sugar levels. In this study, significant differences (*p* < 0.05) were observed between treatments during the first 9 days; furthermore, fruits with CS and CS+CEO retained slightly lower titratable acidity (TA) values at the end of storage, attributable to the barrier effect that reduces metabolic activity. The relationship among TA, pH, and soluble solids confirms their association with the maturity stage. Thus, although the coatings did not prevent the decrease in TA, they did help modulate metabolism and delay the degradation of organic acids compared to the control.

#### 3.3.6. Maturity Index

**Figure 7:**
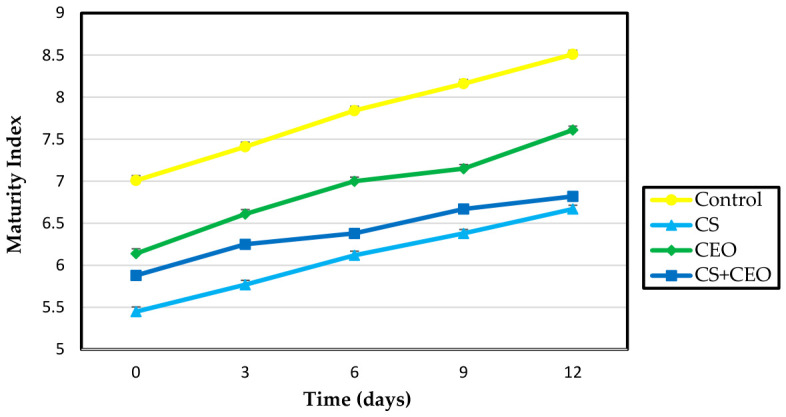
Maturity index of coated topito chili peppers during 12 days of storage at 12 °C ± 2 °C.

The maturity index (MI) of the chili peppers increased progressively across all treatments during storage, reflecting the physiological changes inherent to maturation (Figure 7).

Coated fruits exhibited significantly lower MI values (*p* < 0.05) than the control, indicating that the coatings acted as semipermeable barriers, reducing respiration and transpiration and thereby delaying ripening. The CS emulsion coating exhibited the lowest values across all trials, consistent with other studies indicating that it can extend shelf life by limiting gas and water exchange [74]. Furthermore, the CEO emulsion was observed to reduce the ripening rate of the sweet pepper due to its antimicrobial and antioxidant properties. Similarly, the CS+CEO emulsion exhibited intermediate results, suggesting a complementary effect between the bioactivities of CS and CEO. In this regard, coatings based on these emulsions proved effective in delaying ripening and treating postharvest fungal diseases, highlighting the fundamental role of CS as a sustainable alternative for preserving sweet peppers.

### 3.4. In Situ Antifungal Activity

**Figure 8:**
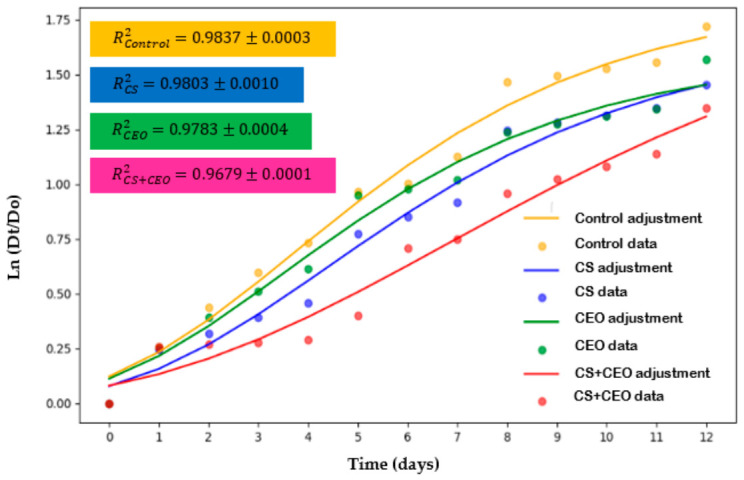
Growth dynamics of *P. expansum* inoculated in topito chili pepper (*C. chinense*) under different treatments.

Figure 8 shows the fit of the modified Gompertz model to radial growth data for the fungus in the fruit incisions, describing the growth dynamics of *P. expansum* under the effects of the different coatings (see Figure S1 in the Supplementary Materials). The model showed a strong fit to the data, validating its use for describing fungal infection in fruit [75,76].

In the control group, the highest growth rate (µ_m_) and a reduced lag phase (λ) were observed, indicating rapid fungal colonization and the absence of barriers limiting its development. Fruits coated with CS showed a significant decrease in µ_m_ and a slight extension of λ, attributable to the formation of a semipermeable film that restricts the entry of nutrients and oxygen [77].

Coating with the CEO emulsion prolonged the lag phase and reduced the growth rate, likely due to the antimicrobial action of eugenol, which destabilizes fungal membranes and induces oxidative stress [78]. Furthermore, the CS+CEO combination proved more effective, showing a greater increase in λ, a lower µ_m_, and a lower A value, indicating a synergistic effect between CS and the phenolic compounds in CEO. This is because CS acts as a controlled-release matrix for bioactive metabolites, strengthening the physical and chemical barriers against fungal colonization. Taken together, the Gompertz model parameters indicate that the coatings delay infection, reduce the growth rate of *P. expansum*, and limit its maximum growth. These results confirm the potential of chitosan and clove essential oil emulsions as a sustainable alternative for postharvest control and shelf-life extension of the topito chili pepper and highlight the usefulness of the Gompertz model for predicting microbial behavior in food.

### 3.5. Microbiological Analyses

**Figure 9:**
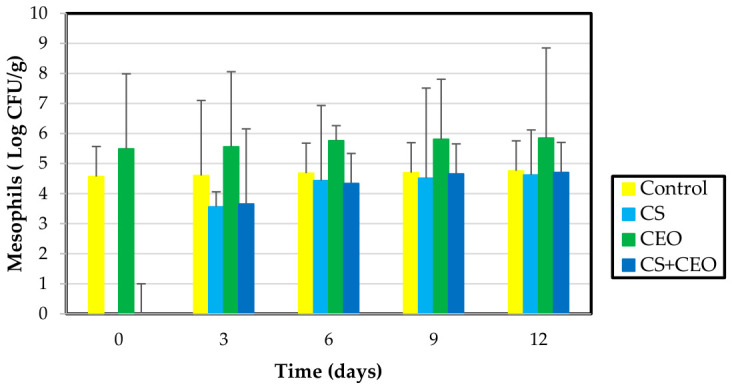
Effect of treatments on the concentration of aerobic mesophiles in topito chili pepper (*C. chinense*) with CS, CEO, and CS+CEO.

In this study, the effect of different coatings (CS, CEO, and their combination (CS+CEO)) on the aerobic mesophilic population of topito chili peppers was evaluated during 12 days of storage at 12 °C ± 2 °C (Figure S2; see Supporting Materials). Aerobic mesophilic bacteria are key indicators of microbial contamination and spoilage, reflecting the fruit’s hygienic quality (Figure 9).

The results obtained showed no significant differences (*p* > 0.05) between treatments or throughout the storage period. On day 0, the samples treated with CEO (5.49 ± 0.35 log CFU/g) showed slightly higher counts than those treated with the CS+CEO combination; the CS and CS+CEO treatments showed lower counts, suggesting a slight initial microbial inhibitory effect, although not statistically significant.

During storage (days 3–12), all treatments maintained similar aerobic mesophilic levels, with no significant variations over time. However, the combined CS+CEO treatment tended to show slightly lower counts (3.65–4.71 log CFU/g) than the control and the CEO-alone treatment, suggesting a possible synergistic effect between chitosan and the phenolic compounds in the essential oil. In general, the application of coatings did not eliminate the microbial population, but it did help to maintain it at stable levels, suggesting a bacterial growth-containment effect. This behavior is consistent with previous reports on fruits coated with biopolymers and essential oils, which have shown a moderate antimicrobial action that helps delay microbial deterioration during storage.

### 3.6. Effect of Coatings on the Sensory Parameters of the Topito Chili Pepper (C. chinense)

Sensory evaluation was conducted by an untrained panel that assessed acceptability attributes, including aroma, color, shine, and texture, of the topito chili pepper. These parameters are essential quality indicators in post-harvest studies, as they directly influence consumer perception and the shelf life of fresh produce.

#### 3.6.1. Aroma

**Figure 10:**
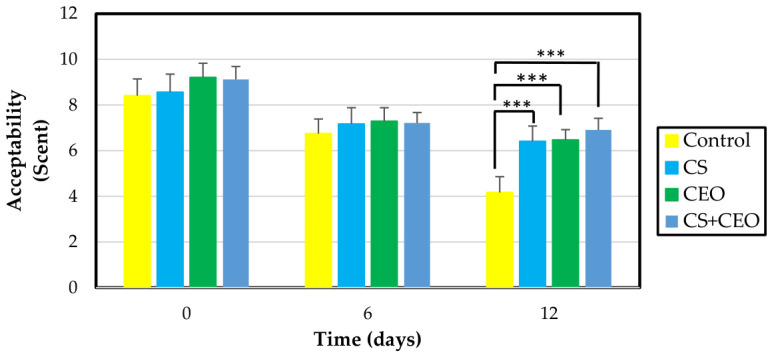
Sensory evaluation of the aroma of topito chili pepper (*C. chinense*) under different treatments. *** Indicates statistically significant differences at *p* < 0.05.

The results show a decrease in aroma acceptability as storage progresses (Figure 10). The control group recorded the lowest score on day 12 (4.17 ± 0.69), whereas the CS, CEO, and CS+CEO treatments showed higher values (6.41 ± 0.67, 6.47 ± 0.45, and 6.91 ± 0.51, respectively). However, the differences between treatments were not statistically significant (*p* > 0.05), although they differed significantly from the control, indicating that all treatments maintained similar acceptability levels.

During the temporal evolution, a significant decrease in aroma score was observed between days 0 and 12, particularly in the control group, indicating a loss of volatile compounds responsible for the characteristic aroma. This indicates that although the coatings (CS, CEO, and CS+CEO) act as barriers to moisture loss and to the loss of certain metabolites, they do not significantly affect the biosynthesis or release of these compounds during ripening. The volatile compounds, mainly apocarotenoids derived from carotenoids, are generated by enzymatic action and, although present in low concentrations, are detectable by sensory analysis. The slightly higher scores in the CEO and CS+CEO treatments could be due to the protective effect of the coatings on these compounds, delaying their degradation and preserving the aroma for longer than in the control.

#### 3.6.2. Gloss

**Figure 11:**
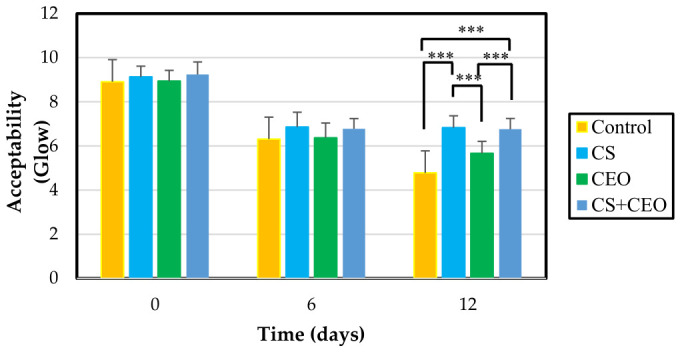
Acceptability analysis of the topito chili pepper (*C. chinense*) under the different treatments. *** Indicates statistically significant differences at *p* < 0.05.

The gloss (Figure 11) reflected physical changes on the fruit surface due to water loss and cuticle alteration. On day 0, all treatments showed high values, with CS+CEO standing out (9.24 ± 0.57), while the control’s value was slightly lower (8.91 ± 0.73). During storage, the gloss rating decreased significantly (*p* < 0.05), especially in the control group, demonstrating that dehydration and surface changes reduce the fruit’s ability to reflect light.

Despite this trend, the coating treatments (CS, CEO, and CS+CEO) showed no significant differences (*p* > 0.05) on days 0 and 6, indicating a moderate effect on gloss preservation, possibly due to the formation of a partial barrier against dehydration and epidermal alterations. Since gloss depends on both light incidence and the evaluator’s perception, its analysis should be complemented with color, as both reflect the fruit’s visual quality. However, on day 12, significant differences (*p* < 0.05) were observed in fruits treated with coatings, especially CS and CS+CEO, which had higher gloss values than the control, which suggests that, with an extended storage time, the effects of the protective coatings are more evident and help to better preserve the fruit’s surface properties. Overall, the coatings partially maintained the chili’s appearance, reducing shine loss without affecting sensory acceptability during storage.

#### 3.6.3. Color

**Figure 12:**
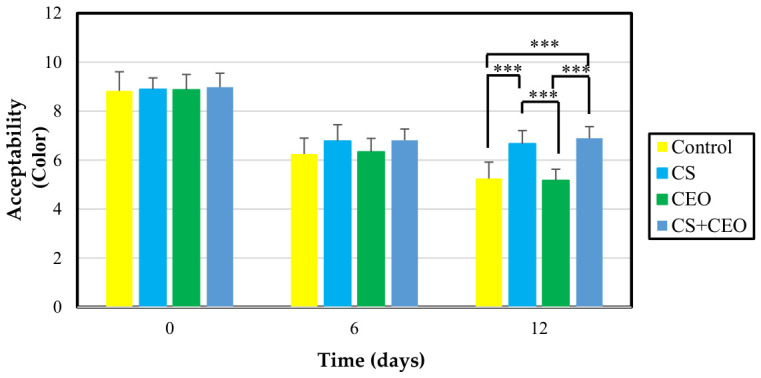
Sensory evaluation of the color of the topito chili pepper (*C. chinense*) under different treatments. *** Indicates statistically significant differences at *p* < 0.05.

The color analysis results (Figure 12) showed a gradual decrease in acceptability during storage. The control group had the lowest score on day 12 (5.25 ± 0.67), while the CS+CEO treatment had the highest (6.89 ± 0.48). However, statistical analysis showed no significant differences (*p* > 0.05) between treatments on days 0 and 6, while significant differences were observed on day 12. The comparative treatments (*p* < 0.05) show that the coatings with CS and CS+CEO have greater color acceptability than the control. From the above, it follows that although this effect was not evident at the start of the test, the coatings more effectively preserved the fruit’s color as storage time increased. This indicates that the coatings significantly influenced the visual perception of the color on day 12.

#### 3.6.4. Texture

**Figure 13:**
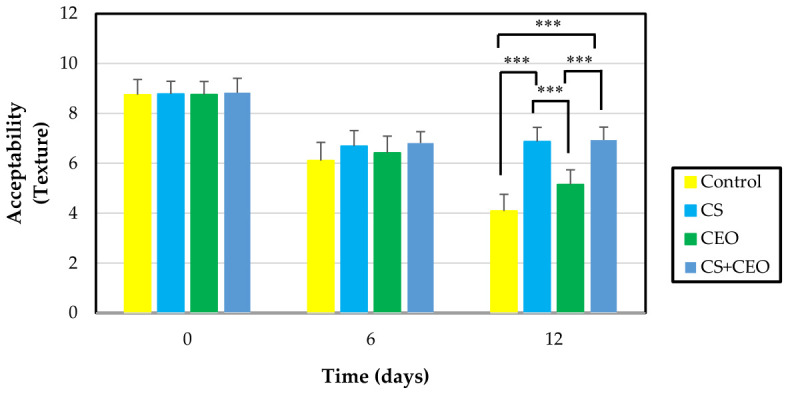
Sensory evaluation of the texture of the topito chili pepper (*C. chinense*) under different treatments. *** Indicates statistically significant differences at *p* < 0.05.

The texture assessment (Figure 13) showed a progressive decrease in acceptability during storage, reflecting the physicochemical changes that the fruit undergoes, such as reduced firmness and water loss.

On day 0, all treatments showed similar and high values (Control: 8.75 ± 0.61; CS+CEO: 8.83 ± 0.58). However, as storage progressed, the texture of the control decreased significantly, reaching 4.09 ± 0.67 on day 12, whereas the coated fruits (CS, CEO, and CS+CEO) maintained higher scores (6.87 ± 0.57, 5.15 ± 0.59, and 6.93 ± 0.52, respectively). Significant differences (*p* < 0.05) were observed on day 12, with the CS+CEO treatment showing the best results, suggesting a synergistic effect in reducing water loss and delaying cell degradation. Overall, the coatings preserved the sensory quality of the topito chili pepper by preserving its texture and mitigating natural softening. This effect is due to the formation of a semi-permeable barrier on the fruit surface, which regulates gas exchange and water loss, reducing the rate of transpiration and respiration, key physiological processes in fruit tissue degradation [71]. By limiting oxygen diffusion into the fruit, coatings can also reduce ethylene production, a hormone that accelerates ripening and cell wall degradation, thereby helping delay softening. The treatments with the least effect on sensory preservation were the control and clove essential oil CEO; the control had the lowest overall acceptability on day 12 due to water loss, which negatively affected the fruit’s color, shine, and texture.

The results of the sensory analysis coincide with those reported by Martínez et al. [49], who observed alterations in texture and color when applying CS and thyme (*Thymus capitatus*) essential oil coatings to strawberries. In this study, edible coatings, especially CS and CS+CEO, helped preserve the sensory acceptability of the topito chili pepper during storage, thereby extending its shelf life compared to the control. These results are consistent with previous research highlighting the synergistic action of CS and essential oils in forming a barrier against moisture loss, visual deterioration, and microbial contamination, thereby maintaining the fruit’s organoleptic quality.

## 4. Conclusions

In this study, edible coatings based on CS and clove essential oil (CEO; CS, CEO, and CS+CEO) were developed and evaluated to extend the shelf life of topito chili pepper (*C. chinense*), which has an approximately 12-day shelf life. With the application of these coatings, especially those formulated with CS and CS+CEO, the shelf life of the topito chili pepper was increased up to 12 days at 12 °C. Clove essential oil showed high levels of eugenol and its derivatives, which have recognized biological properties, supporting its potential as a natural antimicrobial and antifungal agent. The treated fruits, especially those coated with CS and clove essential oil (CS+CEO), exhibited lower weight loss, greater firmness, and improved pH and acidity stability, demonstrating a barrier effect that reduces transpiration and delays ripening.

Furthermore, the coatings exerted a slight inhibitory effect on aerobic mesophiles and on infection of the fruits by *P. expansum*, suggesting a synergistic action between CS and the phenolic compounds of the essential oil. Sensory analysis of the coated fruits confirmed that the coatings maintained acceptable aroma, color, shine, and texture, preserving fruit quality through their physical and microbial barrier properties. All the results presented demonstrate that coatings based on CS and clove essential oil constitute a sustainable and effective alternative for the post-harvest handling of the topito chili pepper.

## Figures and Tables

**Table 1:** Chemical composition of the clove essential oil (*E. caryophyllata*).

Chemical Group	Compound	Kovats Index (Experimental)	Kovats Index (Literature)	Retention Times (RT)	Relative Amount (%)
Esters	Heptanyl-2-acetate	1038	1045 [45]	20.6	tr.
	Eugenyl acetate	1519	1521 [46]	38.5	17.8
Aromatic ester	Methyl salicylate	1197	1192 [47]	26.9	0.2
Monoterpenes (hydrocarbons)	α-Pinene	935	932 [47]	16.1	tr.
	Δ^3^ -Careno	1011	1008 [48]	19.4	tr.
Ketones	2-Nonanone	1091	1092 [44]	22.8	tr.
Oxides	Caryophyllene oxide	1596	1582 [46]	40.8	0.5
Sesquiterpenes (hydrocarbons)	α-Copaene	1384	1374 [47]	33.8	0.1
	trans-β-Caryophyllene	1432	1419 [25]	35.5	4.0
	α-humulene	1468	1454 [47]	36.8	0.5
	δ-Cadinene	1526	1522 [44]	38.7	0.1
	11,11-Dimethyl-4,8-dimethylenebicyl [7.2.0] undecane	1651	1646 [44]	42.3	0.1
Phenols	Chavicol	1252	1247 [44]	28.9	0.2
	Eugenol	1362	1356 [47]	33.0	76.5
	Methyl eugenol	1400	1402 [48]	34.4	tr.

tr.: trace amount (compound detected in trace quantities; relative abundance below 0.1%).

**Table 2:** Physicochemical properties of CS, CEO, and CS+CEO emulsions.

Treatment	pH	ρ (g/cm^3^)	Viscosity (cP)
CS	5.36 ± 0.03 ^a^	1.0087 ± 0.0003 ^a^	32.9 ± 0.40 ^a^
CEO	5.42 ± 0.02 ^b^	1.0043 ± 0.0002 ^a^	2.75 ± 0.12 ^b^
CS+CEO	5.40 ± 0.03 ^c^	1.0074 ± 0.0002 ^a^	32.7 ± 0.26 ^a^

Note: The superscripts (a–c) in the same column refer to significant differences (*p* < 0.05) between treatments.

**Table 3:** Effect of treatments on soluble solids content (°Bx) during the storage stage.

Days	Control	CS	CEO	CS+CEO
0	4.00 ± 0.00 ^a^	3.00 ± 0.00 ^c^	3.50 ± 0.71 ^a^	3.00 ± 0.00 ^c^
3	4.00 ± 0.00 ^a^	3.00 ± 0.00 ^c^	3.50 ± 0.71 ^a^	3.00 ± 0.00 ^c^
6	4.00 ± 0.00 ^a^	3.00 ± 0.00 ^c^	3.50 ± 0.71 ^a^	3.00 ± 0.00 ^c^
9	4.00 ± 0.00 ^a^	3.00 ± 0.00 ^c^	3.50 ± 0.71 ^a^	3.00 ± 0.00 ^c^
12	4.00 ± 0.00 ^a^	3.00 ± 0.00 ^c^	3.50 ± 0.71 ^a^	3.00 ± 0.00 ^c^

Note: The superscripts (a–c) in the same column refer to significant differences (*p* < 0.05) between treatments.

## Data Availability

The data presented in this study are openly available in the article.

## Supplementary Materials

The following supporting information can be downloaded at: https://www.mdpi.com/article/10.3390/polym18121552/s1, Figure S1. In situ antifungal activity in topito chili peppers inoculated with *P. expansum* for 12 days. Figure S2. Aerobic mesophilic counts on Petri dishes for the different treatments (Control, CS, CEO, and CS+CEO).
