# Supplementary material for: Synergistic Coatings Based on Chitosan and Eugenia caryophyllata Essential Oil to Improve Postharvest Quality of Capsicum chinense
Source: Polymers (Basel). 2026 Jun 22;18(12):1552. doi: 10.3390/polym18121552 (PMC13307513; doi:10.3390/polym18121552)
Supplement: Supplementary file 1 [file polymers-18-01552-s001.zip › polymers-4338552-supplementary.pdf]

Supporting information of

Chitosan-Based Coating and Clove Essential Oil (*Eugenia caryophyllata*) for the Preservation of Sweet Chili Paper, Variety Topito (*Capsicum chinense*)

Fanor David Reyes Pérez [1], Yeimmy Peralta-Ruiz [2], Domingo César Carrascal-Hernández [3,*], Johannes Delgado Ospina [4], Clemencia Chaves-López [5] and Carlos David Grande-Tovar [1,*]

[1] Grupo de Investigación de Fotoquímica y Fotobiología, Universidad del Atlántico, Carrera 30 Número 8-49, Puerto Colombia 081008, Colombia; fdreyes@mail.uniatlantico.edu.co; fdreyes@mail.uniatlantico.edu.co

[2] Programa de Ingeniería Agroindustrial, Facultad de Ingeniería, Universidad del Atlántico, Carrera 30 Número 8-49, Puerto Colombia 081008, Colombia; yeimmyperalta@mail.uniatlantico.edu.co

[3] Grupo de Investigación en Sociedad, Educación y Desarrollo Humano GISEDH, Facultad de Ciencias, Educación, Artes y Humanidades, Institución Universitaria de Barranquilla (IUB), Barranquilla 080002, Colombia; dccarrascal@unibarranquilla.edu.co

[4] Grupo de Investigación Biotecnología, Facultad de Ingeniería, Universidad de San Buenaventura Cali, Carrera 122 # 6-65, Cali 76001, Colombia.

[5] Faculty of Bioscience and Technology for Food, Agriculture and Environment, University of Teramo, Via R. Balzarini 1, 64100 Teramo, Italy

* Correspondence: dccarrascal@unibarranquilla.edu.co (D.C.C.-H.); carlosgrande@mail.uniatlantico.edu.co (C.D.G.-T).

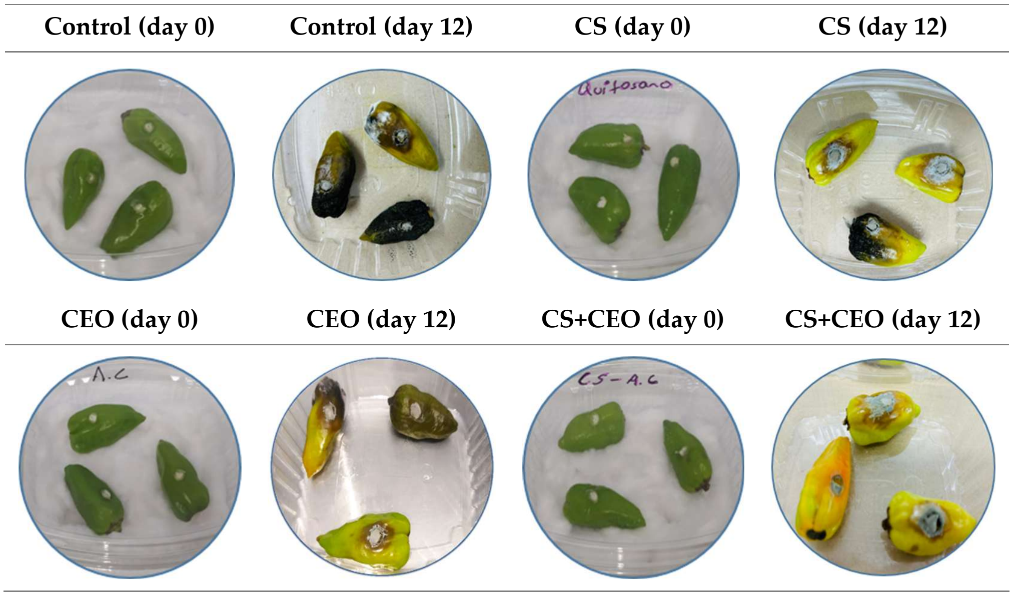

Figure S1. In situ antifungal activity in topito chili peppers inoculated with *P. expansum* for 12 days.

Control	CS
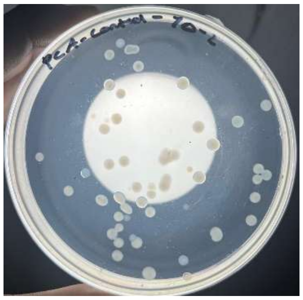	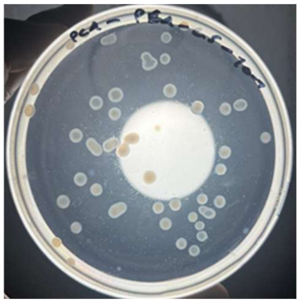
**CEO**	**CS+CEO**
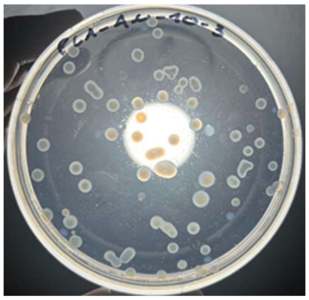	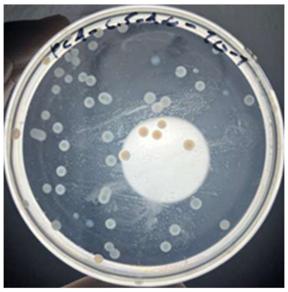

Figure S2. Aerobic mesophil count on Petri dishes for the different treatments (Control, CS, CEO, and CS+CEO).
